# The potential use of bromelain as a natural oral medicine having anticarcinogenic activities

**DOI:** 10.1002/fsn3.999

**Published:** 2019-04-01

**Authors:** Jung‐Ha Lee, Jin‐Tae Lee, Hae‐Ryoun Park, Jin‐Bom Kim

**Affiliations:** ^1^ Department of Preventive and Community Dentistry, School of Dentistry Pusan National University Yangsan Korea; ^2^ Department of Cosmeceutical Science Daegu Haany University Daegu Korea; ^3^ Department of Oral Pathology, School of Dentistry Pusan National University Yangsan Korea; ^4^ BK21 PLUS Project, School of Dentistry Pusan National University Yangsan Korea

**Keywords:** anticarcinogenic effect, bromelain, oral squamous cell carcinoma, public oral health

## Abstract

Bromelain (BR), a protease extracted from *Ananas comosus*, reportedly possesses pharmacological activities including the reduction of thrombogenesis, and antihypertensive, and antimicrobial effects. This study aimed to investigate the potential effects of BR on oral cancer cells. The effect of BR on the viability of Ca9‐22 and SCC25 cells was determined using the MTT assay. These cells were also treated with different doses of BR, and Western blotting was conducted to monitor apoptosis. Finally, flow cytometry analysis was performed to identify sub‐G1 populations of oral cancer cells. After treatment, the viability of both Ca9‐22 and SCC25 cells was markedly reduced, in a dose‐dependent manner. BR induced poly (ADP‐ribose) polymerase (PARP) and lamin A/C degradation, and generated cleavage products. Flow cytometry analysis showed that BR treatment significantly increased the sub‐G1 population. Our findings therefore indicate that BR has potential as a novel, natural anticarcinogenic medicine.

## 1 INTRODUCTION

*Ananas comosus* has been used for centuries by the indigenous inhabitants of Central and South America to treat a range of ailments. Its medicinal qualities are attributed to bromelain (BR), which is extracted from *A. comosus* and has been available as a pharmaceutical product since 1956 (Taussig & Batkin, 1988). BR has recently been used clinically to treat various maladies including edema, thrombophlebitis, sinusitis, inflammation, rheumatic arthritis, and as adjuvant in cancer treatment (Yauan, Wahlqvist, He, Yang, & Li, 2006). BR is a bioactive agent possessing remarkable therapeutic properties and is a complex natural mixture of plant cysteine proteases.

Existing evidence indicates that BR may be a promising candidate for the development of oral enzyme therapies for oncology patients. Adjuvant therapy with external proteases has previously produced positive results in treating cancer, alleviating side effects, and prolonging survival (Zänker, 2001). Earlier reports highlighted the proapoptotic properties of BR in mouse skin tumors and breast cancer cells (Bhui, Prasad, George, & Shukla, 2009). BR acts as an immune modulator by enhancing the impaired immune‐mediated cytotoxicity of monocytes against patient tumor cells (Maurer, 2001) and was shown to possess anticancer properties in hairless mouse skin (Goldstein, Taussig, Gallup, & Koto, 1975).

Oral squamous cell carcinoma (OSCC) is a significant global public health problem, causing high morbidity and mortality with no improvement in decades. Oral cancer is a disfiguring cancer that may lead to facial distortion. OSCC, the most common cancer of the oral cavity, is mainly treated by surgery alone. Because surgical treatment of OSCC sacrifices both important functionality and esthetics of the head and neck, it should be minimized or replaced with other treatment modalities such as chemotherapy and radiotherapy. However, in OSCC, chemotherapy is used as an adjuvant rather than as a single treatment modality as it rarely results in a cure and has limited effectiveness (Deng, Sambrook, & Logan, 2011). Improving OSCC responsiveness to chemotherapeutic agents is essential for patients to obtain more functional and cosmetic results from treatment and could be achieved by deepening our understanding of the mechanisms involved in the low sensitivity of OSCC cells to chemotherapeutic agents (Woo et al., 2017). Consequently, the development of new anti‐oral cancer medicines is very important to improve treatment of OSCC. In the present study, various experiments were performed to determine how BR causes apoptosis in oral cancer cells.

Apoptosis was first described by Kerr, Wyllie, and Currie (1972) and was shown to accompany ischemia of living tissue. In cancer therapy, it occurs in antitumor responses and is a valuable marker for predicting tumor response following anticancer treatment. Apoptosis emphasizes the physiological aspects of the death of individual cells rather than that of a continuous tissue region or cell population. In the early stages of apoptosis, DNA rapidly breaks down, causing chromatin aggregation in the periphery of the nucleus, and a “step ladder pattern” in gel electrophoretic tests. In later stages, changes in the organelles and the disappearance of the cytoplasmic membrane are observed. Apoptotic cell death may result in morphological changes such as membrane blebs, cell shrinkage, chromatin condensation, and nuclear fragmentation with formation of apoptotic bodies. It is characterized by the disappearance of the cytoplasmic membrane and peripheralization of organelles, resulting in cellular edema and nuclear swelling, accumulation of macrophages, and DNA fragmentation as a delayed response. Medical research aims to manipulate the machinery of cell death, and the regulation of apoptosis may lead to new possibilities for oral cancer treatment (Tamatani et al., 2007). Thus, this study aims to demonstrate the apoptotic effects of BR on SCC25 and Ca9‐22 cells.

The purpose of this study was to investigate apoptosis via various experimental methods by treating the oral cancer cells Ca9‐22 and SCC25 with various concentrations of BR. Furthermore, we would like to prove that BR can be an effective new natural medicine for oral cancer.

## 2 MATERIALS AND METHODS

### 2.1 Cell line, antibodies, and chemical reagents

SCC25 human oral squamous carcinoma, HaCaT human keratinocyte, and human gingival fibroblast (HGF‐1) cells were purchased from the ATCC (Rockville, MD, USA). Ca9‐22 human oral squamous carcinoma cells were obtained from the Department of Oral Pathology (Pusan National University, Yangsan, Republic of Korea). 3‐[4,5‐dimethylthiazol‐2‐yl]‐2.5‐diphenyltetrazolium bromide (MTT), acrylamide, and Hoechst33342 were purchased from Sigma Chemical Co. (St Louis, MO, USA). Rabbit polyclonal anti‐human apoptosis‐inducing factor (AIF) antibody was purchased from Upstate (NY, USA), while mouse monoclonal anti‐human caspase‐9, caspase‐7, caspase‐3, BAX, Bcl‐2, cytochrome‐C, lamin A/C, DFF45 (ICAD), poly (ADP‐ribose) polymerase (PARP) antibodies, β‐actin, and rabbit polyclonal anti‐human DFF40 (CAD) antibody were obtained from Stressgen (Ann Arbor, MI, USA).

### 2.2 Preparation and purification of extract from *Ananas comosus* stem and bark

The stem and bark of *A. comosus* were separated from the fleshy fruit, cut into small pieces, and dried. Ethanol (70%, v/v) was then added to elute the enzyme over 48 hr. The supernatant was collected after centrifugation. Ammonium sulfate (70% saturated) was added to coagulate and precipitate the enzyme, and the solution was centrifuged to prepare the crude extract. Purification of the crude extract from *A. comosus* was performed by dialysis with 70% (v/v) ethanol before elution on a DEAE‐cellulose column at a flow rate of 0.7 ml/min with a linear gradient of 0 to 1 M NaCl. The active fractions were resuspended in a G‐150 column at 4 ml/tube and a flow rate of 0.4 ml/min, and were collected, concentrated, and lyophilized. Purified BR was stored at −18°C and was prepared at a concentration of 1,000 μg/ml in distilled water fresh before each experiment.

### 2.3 Anticarcinogenic effects

#### 2.3.1 Cell viability assay

SCC25, HaCaT, HGF‐1, and Ca9‐22 cells were plated at 3 x 10^5 ^cells/mL in 96‐well plates, preincubated for 24 and 48 hr at 37°C, and maintained in a humidified atmosphere containing 5% CO_2_. For all experiments, cells were grown to 80%–90% confluence and subjected to no more than 20 cell passages. Cells were seeded in 96‐well plates, and the viability was determined via MTT assay (Carmichael, DeGraff, Gazdar, Minna, & Mitchell, 1987). The absorbance of converted dye was measured at a wavelength of 550 nm.

#### 2.3.2 Western blotting analysis

Cells were plated at a density of 2 × 10^6^ cells in culture dishes. Cells treated with BR were washed with phosphate‐buffered saline (PBS) and centrifuged. Protein extracts were prepared in extraction buffer. Protein samples from treated and untreated cell extracts were electro‐blotted onto a nitrocellulose membrane. The membrane was incubated overnight at 4°C with primary antibody. The secondary antibody was added and incubated for 2 hr. After washing the membrane with Tris‐buffered saline containing Tween (TBST), the membrane was developed with an electro‐chemiluminescence solution and visualized using a CCD camera system.

#### 2.3.3 Hoechst staining

Hoechst staining was employed for the identification of apoptotic nuclei. After treatment with BR, cells were harvested, centrifuged, and stained with 4 μg/ml Hoechst for 10 min at 37°C. Samples were visualized and photographed under an epifluorescence microscope (Carl Zeiss, Göettingen, Germany).

#### 2.3.4 Flow cytometry analysis

Cells were seeded in a 6‐well plate at 10^6^ cells/ml and incubated. Harvested cells were washed with PBS and centrifuged at 2,000 *g* for 10 min. Fixed cells were pelleted and washed in 1% bovine serum albumin/PBS solution. Cells were resuspended in 1 ml PBS containing 20 μg/ml RNase A, incubated at 4°C for 30 min, and resuspended in propidium iodide (PI) solution. After incubation, the cells' DNA content was measured on a flow cytometer (Beckman Coulter, FL, CA, USA), and the data were analyzed using the software MultiCycle.

### 2.4 Statistical analysis

The data in this report are representative of three or more experiments and are expressed as the means ± standard error of the mean. Differences between means were tested for significance by one‐way analysis of variance using SPSS (version 22.0; SPSS Inc., Chicago, IL, USA). *p* < 0.05 was considered statistically significant.

## 3 RESULTS AND DISCUSSIONS

### 3.1 Purification of extracted bromelain

*Ananas comosus* extracts were dialyzed with 70% ethanol and concentrated. After ion‐exchange chromatography using a DEAE‐cellulose column, five fractions were collected, and the active protein eluted with approximately 0.5 M NaCl (yield approximately 57%). The active fractions were gel filtrated on a G‐150 column to measure enzyme activity, collected, concentrated, and lyophilized. The yield was then approximately 49% and purity was approximately 17‐fold higher than that reported by Silverstein (14.2‐fold; Silverstein & Kezdy, 1975). Purified BR was stored at −18°C and prepared as a 1,000 μg/ml solution in distilled water fresh before each experiment.

### 3.2 Anticarcinogenic effects

#### 3.2.1 Cell viability assay

**Figure 1:**
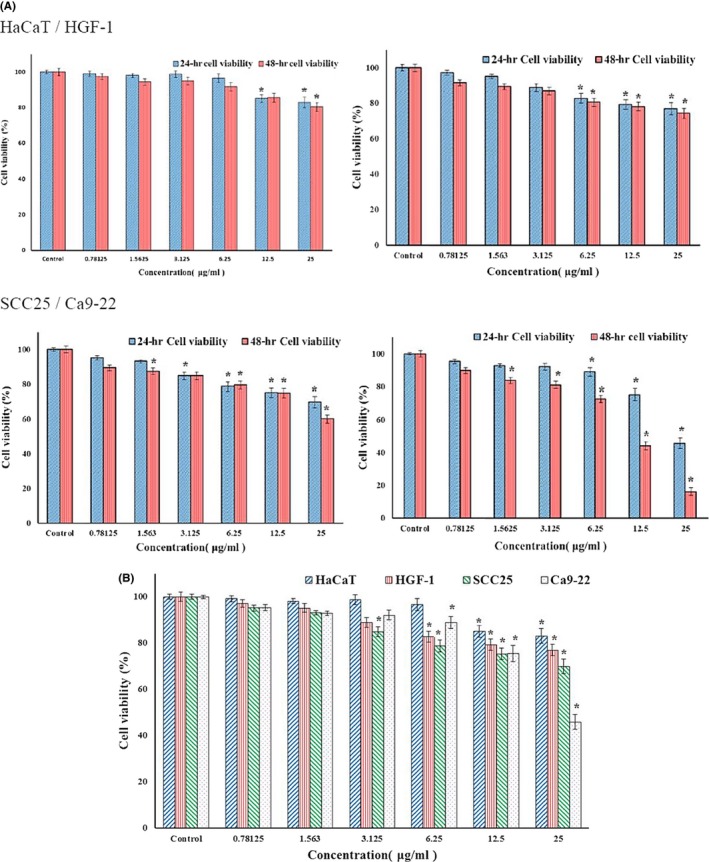
(A) Viability of Ca9‐22, SCC25, and HACAT cells measured via MTT assay and shown after treatment with different concentrations of bromelain (BR) for 24 and 48 hr. (B) Viability of HaCaT, HGF‐1, SCC25, and Ca9‐22 cells measured via MTT assay and shown after treatment with different concentrations of BR for 24 hr. Viability was markedly reduced in Ca9‐22 and SCC25 cells, in a dose‐dependent manner. Ca9‐22 and SCC25 cells were more susceptible to BR than HaCaT cells, and Ca9‐22 cells more so than SCC25 cells. *p < *0.05, compared to the control. Values represent means ± *SD* of independent experiments performed in triplicate

Using the cell viability assay, we investigated the cytotoxic potential of BR by comparing the growth of SCC25 and Ca9‐22 cells with that of HaCaT and HGF‐1 cells. As shown in Figure 1A, the viability of SCC25 and Ca9‐22 cells decreased in a dose‐ and time‐dependent manner after 24‐hr treatment with BR, from 95.16% and 95.24% (at 0.78125 μg/ml) to 69.93% and 49.82% (at 25 μg/ml), respectively. However, the viabilities of HaCaT and HGF‐1 cells were sustained after 24 and 48 hr. As shown in Figure 1B, the viabilities of Ca9‐22 and SCC25 cells decreased markedly in a dose‐dependent manner. Ca9‐22 and SCC25 cells were more susceptible to BR than HaCaT and HGF‐1 cells, and Ca9‐22 cells more so than SCC25 cells.

As shown in Table 1, the viabilities of HaCaT, HGF‐1, SCC25, and Ca9‐22 cells were 83.07%, 76.93%, 69.94%, and 45.28%, respectively, when treated with 25 μg/ml BR. The half maximal inhibitory concentrations (IC_50_) of BR were calculated from concentration‐response curves plotting growth percentage.

**Table 1:** Viability of HaCaT, HGF‐1, SCC25, and Ca9‐22 cells treated for 24 hr with the indicated concentrations of bromelain

Cell	Hacat		HGF‐1		SCC25		Ca9‐22	
	Cell viability (%) at 25 μg/ml	IC_50_ value (μg/ml)	Cell viability (%) at 25 μg/ml	IC_50_ value (μg/ml)	Cell viability (%) at 25 μg/ml	IC_50_ value (μg/ml)	Cell viability (%) at 25 μg/ml	IC_50_ value (μg/ml)
Treatment	83.07 ± 0.9174	198.4 ± 1.407	76.93 ± 0.6801^a^	73.78 ± 0.903^a^	69.94 ± 0.9762^a^	33.02 ± 0.687^a^	45.82 ± 1.044^a^	14.07 ± 0.324^a^
Bromelain								

Notes

All data are expressed as mean ± *SD* (*n* = 3). The letter a in each column denotes statistical significance compared to the control group (HaCat cells) at *p < *0.05

HGF‐1: human gingival fibroblast.

#### 3.2.2 Morphological analysis of Ca9‐22 and SCC25 cells treated with bromelain at different concentrations

**Figure 2:**
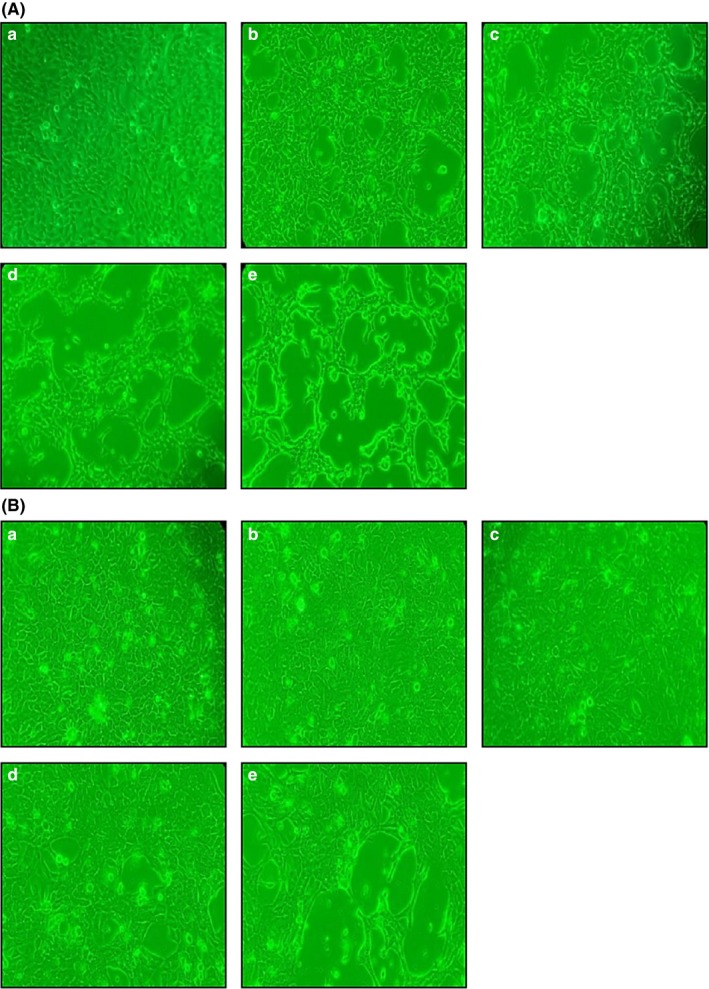
Morphological characteristics of Ca9‐22 (A) and SCC25 (B) cells treated with different concentrations of bromelain (BR) for 24 hr. (a) Control (no treatment), (b) 3.12 μg/ml, (c) 6.25 μg/ml, (d) 12.5 μg/ml, and (e) 25.0 μg/ml BR

Treatment of Ca9‐22 and SCC25 cells with different concentrations of bromelain for 24 hr resulted in apoptosis‐associated morphological changes (Figure 2). This image results displayed characteristic apoptotic changes such as condensed chromatin, cytoplasmic membrane blebs, and apoptotic bodies. However, HGF‐1 and HaCaT cells showed amoeboid, spindle‐shaped and cobblestone pattern morphology of normal cells. This indicates that even a low concentration of bromelain prevented cell growth and induced aggregation of existing cells in Ca9‐22 and SCC25 cells.

#### 3.2.3 Western blotting analysis

**Figure 3:**
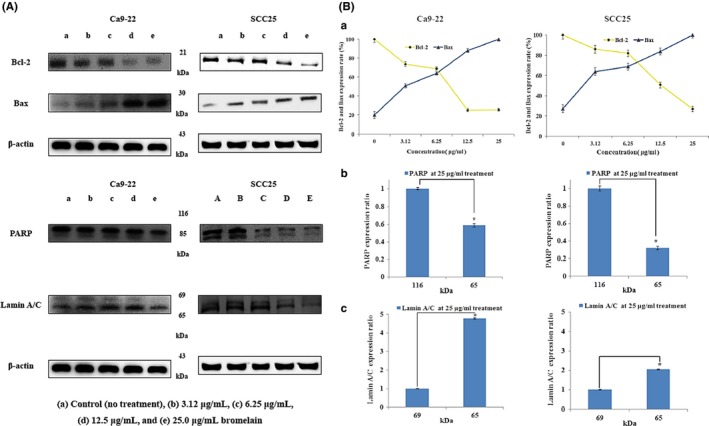
Effects of bromelain (BR) on the expression of apoptosis regulators. Ca9‐22 and SCC25 cells were treated with different concentrations of BR for 24 hr. (A) The expression of Bcl‐2, BAX, PARP, and lamin A/C were determined via Western blot analysis. BR induced PARP and the cleaved form of lamin A/C, and produced 86 kDa PARP and 116 kDa lamin A/C cleavage products. (B) Expression of BAX increased compared to that in the control in a dose‐dependent manner, and expression of Bcl‐2 decreased. β‐Actin levels were used as an internal standard to quantify Bcl‐2, BAX, PARP, and lamin A/C expression. (a) Bcl‐2 and BAX expression ratio (criteria = 1), (b, c) lamin A/C and PARP expression ratio (criteria = 1) when treated with 25 μg/ml BR. *p < *0.05, compared to the control. Data are expressed as means ± *SD* of three independent experiments. PARP: poly ADP‐ribose polymerase

Mitochondrial pathways can be triggered by various intra‐ and extracellular stress signals, resulting in activation of proapoptotic proteins, including BAX, or inactivation of antiapoptotic Bcl‐2 family members, such as Bcl‐2 (Orrenius, 2004). Induction of apoptosis is regulated by Bcl‐2 family members, and Bcl‐2 is antiapoptotic, whereas BAX promotes apoptosis. To examine the role of Bcl‐2 family proteins in BR‐induced apoptosis, Western blotting was performed. As shown in Figure 3A, expression of BAX increased following BR treatment and Bcl‐2 was down‐regulated in a dose‐dependent manner. These results indicate that a shift in the expression ratio of BAX to Bcl‐2 may be the molecular mechanism by which BR induces apoptosis in Ca9‐22 and SCC25 cells. Furthermore, we examined the effects of BR on PARP and lamin A/C via Western blotting. As shown in Figure 3A, BR treatment induced PARP and lamin A/C degradation, and generated PARP 85 kDa and lamin A/C 65 kDa.

**Figure 4:**
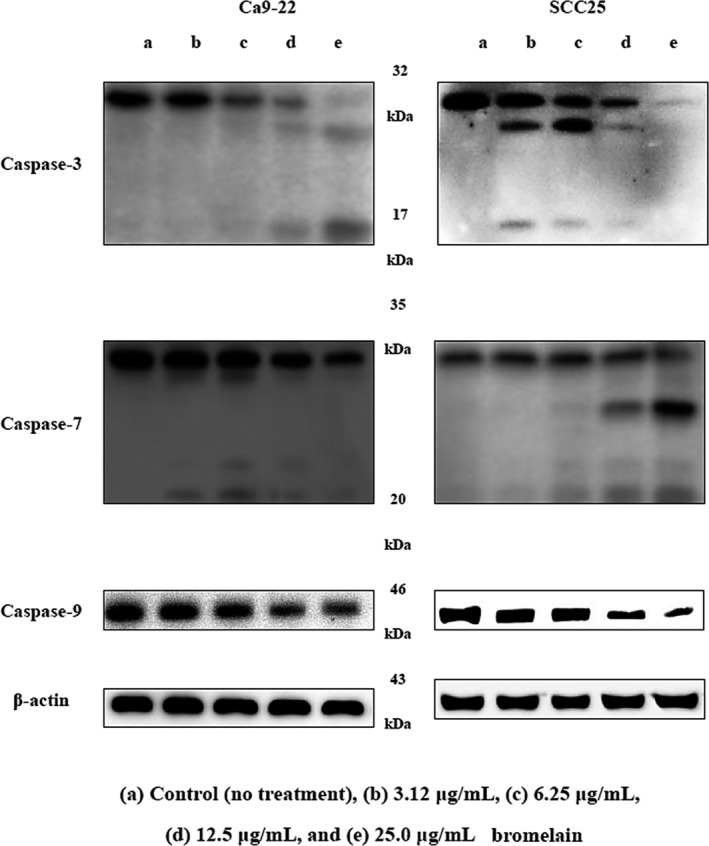
Effects of bromelain (BR) on the expression of apoptosis regulators. Ca9‐22 and SCC25 cells were treated with different concentrations of BR for 24 hr. The expression of caspase‐3, caspase‐7, and caspase‐9 was determined via Western blot analysis

Caspases play an essential role in apoptosis (Acehan et al., 2002). Once activated, effector caspases‐3, caspases‐7, and caspases‐9 are responsible for the proteolytic cleavage of various targets, ultimately leading to cell death (Kim et al., 2011). Previous studies have demonstrated the proapoptotic effects of BR in several in vitro and in vivo cancer models. Kalra et al. (2008) observed induction of caspase‐3 and caspases‐9 after treatment with BR, and Wales et al. observed BR‐related induction of caspase‐dependent apoptosis in MKN45 cells (Amini et al., 2013). In this study, BR induced degradation of caspase‐3 and caspases‐7, and produced 17 kDa caspase‐3 and 20 kDa caspase‐7 cleavage products. Caspase‐9 expression levels decreased compared to those of the control in a dose‐dependent manner (Figure 4). These results therefore indicate that caspase activation is involved in BR‐mediated apoptosis in Ca9‐22 and SCC25 cells.

**Figure 5:**
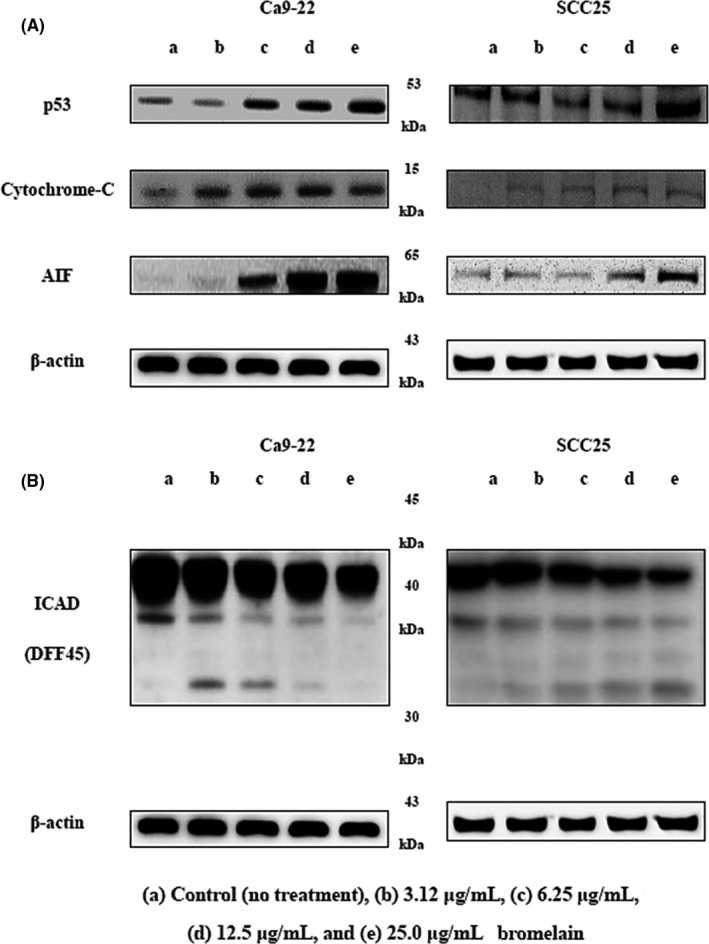
Effects of bromelain (BR) on expression of apoptosis regulators. Ca9‐22 and SCC25 cells were treated with different concentrations of BR for 24 hr. (A) Expression of p53, cytochrome‐C, and AIF, and (B) ICAD (DFF45) determined by Western blot analysis. AIF: apoptosis‐inducing factor; ICAD: inhibitor of caspase‐activated DNase

New evidence suggests a direct, extra‐nuclear, transcription‐independent apoptotic function of p53, in addition to its antitumorigenic role as a sequence‐specific, proapoptotic transcription factor (Chipuk & Green, 2003). Kal et al. demonstrated that DMBA–TPA treatment down‐regulated p53 expression when compared to that in untreated controls. However, BR treatment resulted in upregulation of p53 in comparison with DMBA–TPA treatment (Kalra et al., 2008). As shown in Figure 5A, after BR treatment in our study, expression of p53 increased compared to that in the control in a dose‐dependent manner.

We also investigated BR‐induced release of cytochrome‐C, which contributes to apoptosis triggered by proteasome inhibition, from mitochondria into the cytosol. Our results suggest that BR‐induced apoptosis accompanies cytochrome‐C release (Figure 5A). Changes in the mitochondrial membrane resulting from the activation/inactivation of Bcl‐2 family proteins may lead to dissipation of the inner membrane potential and permeabilization of the outer mitochondrial membrane. The release of various proapoptotic proteins such as cytochrome‐C and AIF is then induced (Barczyk et al., 2005; Brouckaert, Kalai, Saelens, & Vandenabeele, 2005; Hengartner, 2000). AIF expression increased for 24 hr after treatment with BR (Figure 5A). It was released from mitochondria and translocalized into the nuclei in both Ca9‐22 and SCC25 cells. Known cellular substrates include inhibitors of deoxy‐ribonuclease such as DFF45 (ICAD; Porter, 1999). The final stage of apoptosis includes nuclear condensation, controlled by DFF (Kim et al., 2011). Once DFF40 (CAD) is activated and released from the ICAD (DFF45)/DFF40 (CAD) complex, it can translocate to the nucleus where it degrades chromosomal DNA (Cheng et al., 2007). As shown in Figure 5B, BR induced ICAD (DFF45) degradation and produced 30 kDa cleavage products.

#### 3.2.4 Hoechst staining

**Figure 6:**
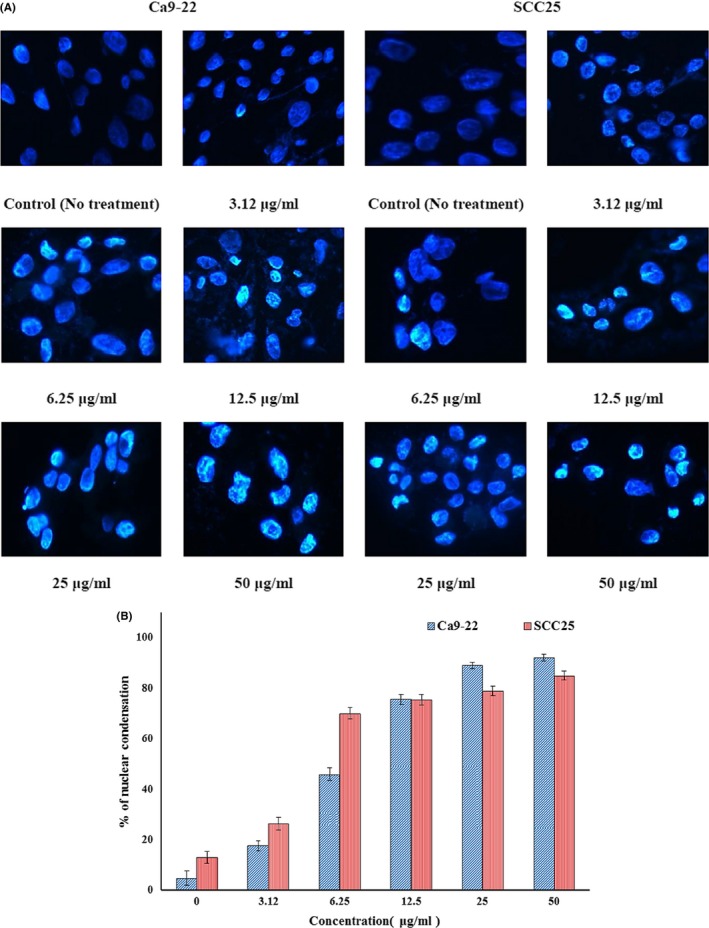
Investigation of apoptosis in Ca9‐22 and SCC25 cells treated with bromelain (BR) for 24 hr. (A) Immunofluorescent micrographs after Hoechst staining. Control cells displayed round nuclei. Cells treated with BR displayed dose‐dependent nuclear condensation. (B) The values below micrographs are the mean numbers (±*SD*) of apoptotic cells, determined by Hoechst staining (*n* = 3)

Hoechst staining revealed BR‐induced changes in nuclear morphology. Untreated cells displayed typical round nuclei, whereas Ca9‐22 and SCC25 cells treated with 3.12 to 50 μg/ml BR for 24 hr displayed condensed and fragmented nuclei (Figure 6A). As in the cell viability experiment, Ca9‐22 cells were more susceptible to BR than SCC25 cells (Figure 6B). Nuclear condensation was 89% and 78% in Ca9‐22 and SCC25 cells, respectively, after treatment with 25 μg/ml BR.

#### 3.2.5 Flow cytometry analysis for cell cycle progression

**Figure 7:**
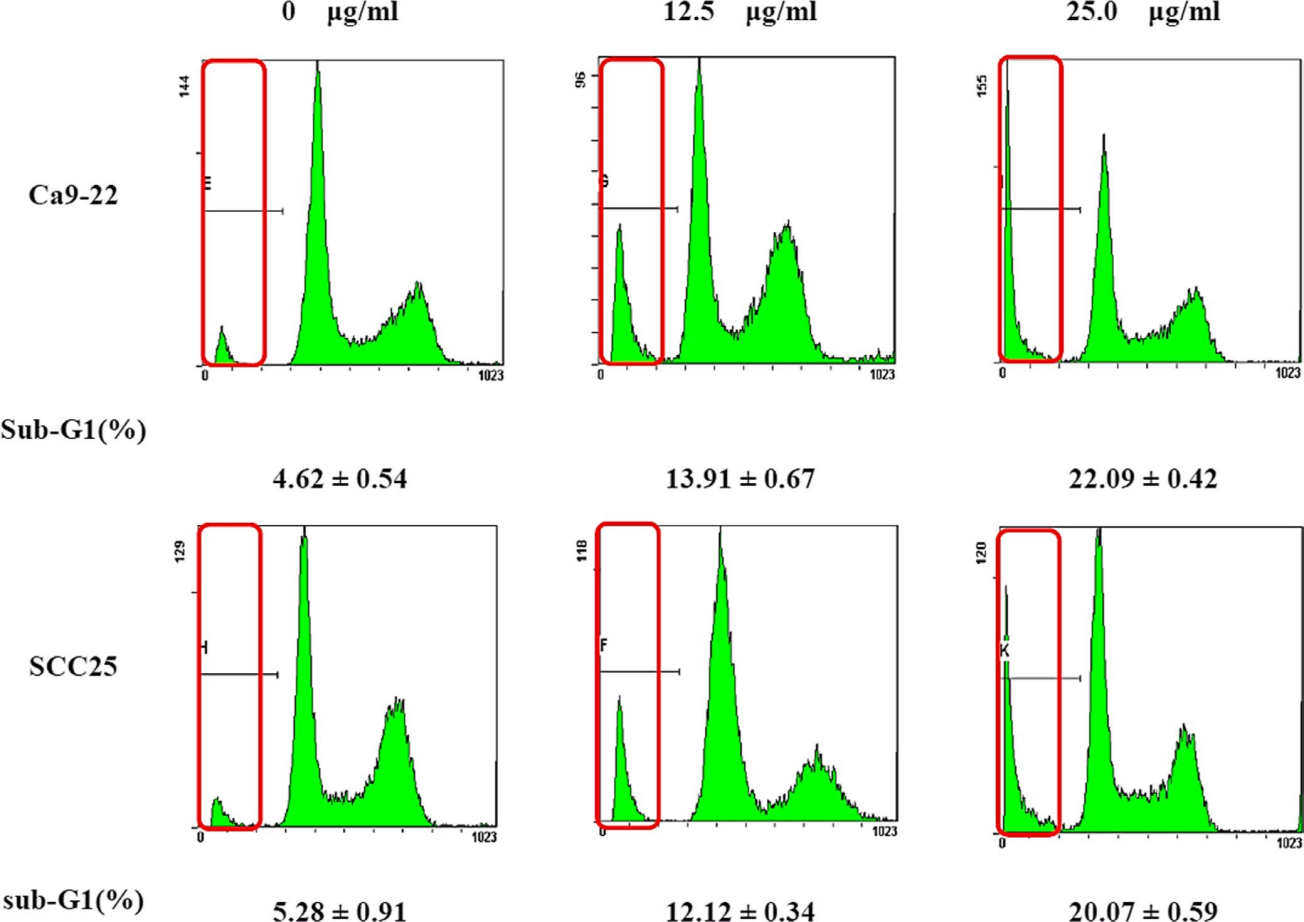
Kinetic analysis of the effects of bromelain (BR) treatment on cell cycle progression in Ca9‐22 and SCC25 cells, and induction of apoptosis, using flow cytometry. Ca9‐22 and SCC25 cells were treated with 0, 12.5, and 25.0 μg/ml BR for 24 hr. Flow cytometry histograms show the distribution of cells in different phases of the cell cycle and the number of cells (*y*‐axis) versus the DNA content (*x*‐axis). Values below are mean percentages (±*SD*) of sub‐G1 stage cells (*n* = 3)

The cell cycle and apoptotic cell percentages were investigated using flow cytometry. Loss of DNA is a typical feature of apoptotic cells that occurs as degraded DNA diffuses out of the cells after endonuclease cleavage. After staining with PI, cells that have lost DNA take up less stain (Kundu, Dey, Roy, Siddiqi, & Bhattacharya, 2005). As shown in Figure 7, BR increased the sub‐G1 population of Ca9‐22 and SCC25 cells in a dose‐dependent manner, while decreasing the percentage of S‐phase cells. The increase in sub‐G1 phase cells and concomitant decrease in S‐phase cells correlated with an increase in apoptotic cell numbers.

Bromelain has been studied for its antithrombotic and its antimetastatic properties. Previous studies showed that BR significantly reduced local tumor growth and experimental lung metastases in mice (Braun, Schneider, & Beuth, 2005), and inhibited metastasis‐associated platelet aggregation and tumor cell invasiveness in the B16F10 murine melanoma cell line (Paroulek, Jaffe, & Rathinavelu, 2010). To evaluate efficacy and bioactivity, toxicity and in vitro experiments should be conducted before in vivo experimentation. In this study, the viabilities of SCC25 and Ca9‐22 cells were reduced in a time‐dependent manner from 95.16% and 95.24% (0.78125 μg/ml BR) to 69.93% and 49.82% (25 μg/ml), respectively. However, the viabilities of HaCaT and HGF‐1 cells were sustained for 48 hr longer than that of SCC25 and Ca9‐22 cells. Amini et al. investigated the potential of BR to inhibit growth of gastrointestinal cancer cells. It significantly inhibited cell proliferation in MKN45 (*p* = 0.0018, 0.0010, 0.0002, and 0.0001 using concentrations of 100, 200, 400, and 600 µg/ml, respectively), KATO‐III (*p* = 0.0001 using concentrations of 100, 200, and 400 µg/ml, respectively), and 5F12 and 5M21 (*p* = 0.0001 using concentrations of 40 and 50 µg/ml, respectively; Amini et al., 2013). BR IC_50_ values were calculated from concentration–response curves plotting growth percentage and were 198.4, 73.78, 33.02, and 14.07 μg/ml in HaCaT, HGF‐1, SCC25, and Ca9‐22 cells, respectively. At this time, this study demonstrated that BR at specific concentrations (25 μg/ml) showed different IC_50_ values targeting to oral cancer cells. In further investigation, it is planned to determine IC_50_ values for various oral cancer cells, including SCC25 and Ca9‐22 oral cancer cells, at various concentrations.

Apoptosis, or programed cell death, maintains the homeostasis of an individual through embryonic development, tissue rearrangement, and removal of damaged and harmful cells. The life cycle of a cell is regulated by active cell death (Grabowska, Eckert, Fichtner, Schulzeforster, & Maurer, 1997). Cellular suicide displays morphological features, such as cell contraction, chromatin condensation, DNA fragmentation, and cell membrane water saturation, and is regulated by several genes.

Bcl2 family proteins inhibit apoptosis, while BAD, BAX, caspase‐3, and Bim promote apoptosis forming homodimers among themselves or heterodimers with apoptosis‐suppressing proteins such as BAX. Cell death by apoptosis is largely due to two mechanisms: caspase‐8, an early caspase, is activated by ligation of death receptors, such as Fas receptor or tumor necrosis factor, in turn activating further caspases. Alternatively, caspase‐9, another early caspase, activates other caspases and leads to apoptosis. Secretion of cytochrome‐C from the mitochondria is required to activate caspase‐9. Secreted cytochrome‐C interacts with Apaf‐1, and Bcl2 family proteins are important regulators in this mechanism. Activation of caspase‐8 or caspase‐9 directly activates caspase‐3, which in turn activates caspase‐1 and caspase‐2. Thus, caspase‐1, caspase‐2, and caspase‐3 are activated by stimulation of two different apoptotic pathways. The Bcl2 protein family promotes or inhibits cytochrome‐C migration into the cytoplasm and regulates sensitivity to apoptosis. This function is enhanced by BAX, an apoptosis‐promoting protein, and is inhibited by Bcl2, an apoptosis‐suppressing protein. Apoptosis is partially dependent on the balance between proteins that mediate cell death, such as BAX, and proteins that promote cell survival like Bcl2. BR treatment increased expression of BAX and down‐regulated Bcl‐2 in a dose‐dependent manner. This result indicates that an increase in BAX:Bcl‐2 expression may be the molecular mechanisms by which BR induces apoptosis of oral cancer cells. Additionally, BR induced caspase‐3 and caspase‐7 degradation and generated cleavage products. Caspase‐9 expression decreased compared to that in the control in a dose‐dependent manner. Therefore, these results indicate that mitochondrial‐mediated caspase activation is involved in BR‐mediated apoptosis in oral cancer cells. Furthermore, we examined the effects of BR on PARP and lamin A/C via Western blotting. BR induced PARP and lamin A/C degradation, and produced PARP 85 kDa and lamin A/C 65 kDa (Figure 3). Sayan, Sayan, Knight, Melino, and Cohen (2006) demonstrated that p53 could be cleaved by caspases, generating two cytosolic fragments that translocate to the mitochondria and induce depolarization of the mitochondrial membrane. In our study, p53 expression increased compared to that in the control in a dose‐dependent manner. Likewise, Engwerda study showed that BR treatment down‐regulated p53 expression (Engwerda, Andrew, Murphy, & Mynott, 2001). Next, we investigated the effects of BR on cytochrome‐C release, as it is known to contribute to proteasome inhibition‐triggered apoptosis (Wagenknecht et al., 2000) and expression of proapoptotic Bcl‐2 proteins (Hunter & Parslow, 1996). In this study, BR‐induced apoptosis was accompanied by modulation of cytochrome‐C release. Nuclear condensation is controlled by DFF, which once DFF40 (CAD) is activated and released from the DFF45 (ICAD)/DFF40 (CAD) complex can translocate to the nucleus where it degrades and fragments chromosomal DNA (Kundu et al., 2005). BR induced ICAD (DFF45) degradation and produced 30 kDa cleavage products.

Ca9‐22 and SCC25 cells treated with BR displayed characteristic apoptotic changes such as condensed chromatin, cytoplasmic membrane blebs, and apoptotic bodies. However, HGF‐1 and HaCaT cells displayed the amoeboid, spindle‐shaped, and cobblestone morphology of normal cells. BR therefore appears to have an effect on human oral squamous carcinoma cells.

By using Hoechst stain to stain cell nuclei to confirm chromatin condensation and cleavage, normal and apoptotic cells can be distinguished (Jiang, Wang, Ganther, & Lu, 2001). Untreated cells had typical round nuclei, whereas Ca9‐22 and SCC25 cells treated with BR displayed condensed and fragmented nuclei.

The cell cycle and apoptotic cell percentages were investigated using flow cytometry. Translocation of membrane phosphatidylserine in the sub‐G1 state is a form of programed cell death that occurs naturally and can be beneficial in cancer therapy (Lopéz et al., 2002). BR treatment significantly increased the number of apoptotic cells with DNA hypo‐ploidy. In this study, BR treatment increased the sub‐G1 population of Ca9‐22 and SCC25 cells, while decreasing the percentage of S‐phase cells.

## 4 CONCLUSIONS

Based on this study, more systematic investigations should be conducted. It is hoped that BR will be developed as an anticarcinogenic medicine through further safe and effective medical clinical studies. In conclusion, the efficacy of BR was studied in Ca9‐22 and SCC25 cells to develop safer and superior anticarcinogenic agents. Treatment with BR inhibited the growth and proliferation of oral cancer cells, and induced apoptosis in Ca9‐22 and SCC25 cells via various pathways and G1 cell cycle arrest. Therefore, BR may be an effective anticancer drug candidate, specifically for oral cancer, and we hope it will help maintain and promote public oral health.

## ETHICAL STATEMENT

This work does not involve any human or animal studies.

## CONFLICT OF INTEREST

The authors declared no conflict of interest.
